# Engineered Polyploid Yeast Strains Enable Efficient Xylose Utilization and Ethanol Production in Corn Hydrolysates

**DOI:** 10.3389/fbioe.2021.655272

**Published:** 2021-03-05

**Authors:** Lulu Liu, Mingjie Jin, Mingtao Huang, Yixuan Zhu, Wenjie Yuan, Yingqian Kang, Meilin Kong, Sajid Ali, Zefang Jia, Zhaoxian Xu, Wei Xiao, Limin Cao

**Affiliations:** ^1^Beijing Key Laboratory of Plant Gene Resources and Biotechnology for Carbon Reduction and Environmental Improvement, College of Life Sciences, Capital Normal University, Beijing, China; ^2^School of Environmental and Biological Engineering, Nanjing University of Science and Technology, Nanjing, China; ^3^School of Food Science and Engineering, South China University of Technology, Guangzhou, China; ^4^School of Bioengineering, Dalian University of Technology, Dalian, China; ^5^Key Laboratory of Environmental Pollution Monitoring and Disease Control, School of Basic Medical Sciences, Guizhou Medical University, Guiyang, China; ^6^Department of Biochemistry, Microbiology and Immunology, University of Saskatchewan, Saskatoon, SK, Canada

**Keywords:** *Saccharomyces cerevisiae*, mutation, triploid, hydrolysate, ethanol

## Abstract

The reported haploid *Saccharomyces cerevisiae* strain F106 can utilize xylose for ethanol production. After a series of *XR* and/or *XDH* mutations were introduced into F106, the *XR-K270R* mutant was found to outperform others. The corresponding haploid, diploid, and triploid strains were then constructed and their fermentation performance was compared. Strains F106-KR and the diploid produced an ethanol yield of 0.45 and 0.48 g/g total sugars, respectively, in simulated corn hydrolysates within 36 h. Using non-detoxicated corncob hydrolysate as the substrate, the ethanol yield with the triploid was approximately sevenfold than that of the diploid at 40°C. After a comprehensive evaluation of growth on corn stover hydrolysates pretreated with diluted acid or alkali and different substrate concentrations, ethanol yields of the triploid strain were consistently higher than those of the diploid using acid-pretreatment. These results demonstrate that the yeast chromosomal copy number is positively correlated with increased ethanol production under our experimental conditions.

## Introduction

The global corn yield has reached 1.1 billion tonnes in 2019, ranking first in all kinds of grain output. Due to their abundance, corn and corn stover have become popular feedstocks for the production of various industrial fuels and chemicals such as bioethanol and D-lactic acid (Wang et al., 2017). Bioethanol produced by microbes can be used as alternative fuel to alleviate the looming energy shortage and current environmental problems caused by fossil fuel combustion (Chen et al., 2018). *Saccharomyces cerevisiae* preferentially ferments glucose, and it cannot naturally metabolize xylose, the second most abundant carbohydrate in plant biomass hydrolysates (Xiong et al., 2013; van Dijk et al., 2019). Therefore, maximizing ethanol production from xylose by metabolic engineering of *S. cerevisiae* has become a research hotspot (Kim et al., 2013).

Three xylose utilization pathways can be introduced to realize xylose catabolism, including the Dahms or Weimberg pathway, the X-1-P or R-1-P pathway and the classical XR-XDH or XI pathway (Li et al., 2019). The XR-XDH branch can support higher metabolic fluxes than the XI branch, which is limited by the low catalytic efficiency of isomerase. However, an unbalanced redox system due to different cofactor preferences of XR and XDH needs to be optimized to reduce xylitol accumulation and increase conversion of xylose to ethanol (Xiong et al., 2011; Jo et al., 2017). The redox balance can be approached by protein engineering, including K270R, K270M and R276H substitutions in XR, or the D207A/I208R/F209S triple substitution in XDH (Kostrzynska et al., 1998; Watanabe et al., 2005; Watanabe et al., 2007a,b).

Meanwhile, it has been reported that polyploidy can accelerate the evolutionary adaptation of yeast, which is often accompanied by chromosomal aneuploidy, concerted chromosome loss, and point mutations (Selmecki et al., 2015). Compared with haploid strains, diploid and triploid strains, including those obtained by mating two haploid strains with suboptimal xylose fermentation phenotypes, showed an improved xylose fermentation ability and increased ethanol yield, in addition to increased tolerance to heat, acid, ethanol and other inhibitors (Chen et al., 2012). Therefore, the development of polyploid strains with modified redox balance may enable a further enhancement of xylose utilization and ethanol production.

With an average annual yield of 10 tons/hectare, corn is one of the major starchy crops widely used for bioethanol production. However, the process requires pretreatment, liquefaction, saccharification, fermentation and distillation to produce ethanol (Chen et al., 2018; Cripwell et al., 2020). The common methods, such as pretreatment with dilute acid (DA), dilute alkali (AL) and ionic liquids, contribute to breaking down the rigid structure of lignin and hemicellulose to release cellulose for the enzymatic hydrolysis, producing sugars that are then converted to ethanol (Huang et al., 2020; Sankaran et al., 2020). Additionally, a promising amino pretreatment method based on urea was found to effectively improve the performance of enzymatic saccharification of corn stover (Wang et al., 2018). Therefore, it is desirable to make full use of corn hydrolysates, including corn flour and corn distiller’s grains, corn stover hydrolysates with/without urea and corncob fermentation broth, for the evaluation of polyploid strains.

In this study, we attempted to address the redox imbalance for improved xylose conversion capability by constructing different XR and/or XDH mutant strains. Previous studies have constructed haploid strains with relevant favorable mutations (Xiong et al., 2013; Jo et al., 2017). Here we constructed diploid and triploid strains based on the above work, and evaluated their fermentation performance in comprehensive corn hydrolysates. Our results demonstrate that an increase of ploidy can improve yeast fermentation efficiency, which lays a ground for the development of yeast-based cellulosic ethanol.

## Materials and Methods

### Construction of Yeast Strains S15 and S16

The *S. cerevisiae* strains and plasmids used in this study are listed in Supplementary Tables 1, 2. To cross yeast strains, equal amount of two mating type yeast cells were mixed in a centrifuge tube (containing aseptic water). A proper amount of mixed liquid was cultivated on YPD solid medium (20 g/L glucose, 20 g/L peptone, 10 g/L yeast extract, 20 g/L agar) at 30°C for 2 days. Single colonies were used to inoculate a sporulation medium (1% potassium acetate, 1.5% agar) and incubated at 28°C for 2–3 days. The diploid strains were identified by observing sporulation and mating-type confirmation by PCR (Tani et al., 1993). The triploid strains were obtained by similar methods, mainly using a mixed culture of diploid (*MAT****a***/***a***) and haploid (*MAT*α) cells, followed by strain confirmation.

### Construction of Plasmids YEp-CAS and YEp-KCAS

Plasmid YEp-CAS has two mutant sites: (1) The DNA sequence of the first one is “TCCAGATTCTCCGACGAATA C” corresponding to position + 286 bp to + 306 bp concerning the ATG start codon of the *XYL2* gene, whose sequence was changed to “TGTAGATTCTGTGACGAATGT”; (2) The DNA sequence of the second one is “GACATTTTCGACAAC” corresponding to position + 619 bp to + 633 bp, covering the ATG start codon of the *XYL2* gene, whose sequence was changed to “GCTAGATCCGACAGA”.

The plasmid YEp-CAS was constructed as follows: (1) The two DNA fragments C4-1 and C4-2 were amplified by PCR from plasmid YEp-3X using the primer pairs XYL2-U/XYL2C4-D and XYL2C4-U/XYL2 KPN-D and digested with *Dpn*I (Supplementary Table 2; Xiong et al., 2011; Xiong et al., 2013). (2) A fragment of approximately 400 bp was amplified by PCR using primers XYL2-U and XYL2-D with C4-1 and C4-2 as templates, digested with *Sal*I and *Xba*I, and inserted into the corresponding sites of plasmid pUC18 to form pUC18-C4. (3) ARS-1 and ARS-2 were amplified by PCR using primer pairs XYL2KPN-U/XYL2 ARS-D and XYL2ARS-U/XYL2-D with plasmid YEp-3X as the template, and digested with *Dpn*I (Supplementary Table 3). (4) A fragment of approximately 700 bp was amplified by PCR using primers XYL2 KPN-U and XYL2-D with ARS-1 and ARS-2 as templates, digested with *Kpn*I and *Xba*I, and inserted into the corresponding sites of plasmid pUC18-C4 to form pUC18-CAS. The resulting plasmid pUC18-CAS was confirmed by sequencing. (5) The mutant *XYL2* gene was cleaved from pUC18-CAS by using *Sal*I and *Xba*I, and inserted into plasmid pUC18-PXYL2 to form pUC18-PXYL2 (CAS). (6) The wild-type sequence of PXYL2 in the plasmid YEp-3X was replaced by the corresponding sequence of pUC18-PXYL2 (CAS) to form plasmid YEp-CAS. YEp-CAS was the shuttle plasmid expressing *XYL1*, *XYL2* (CAS) and *XKS1* (Xiong et al., 2011). To construct the plasmid YEp-KCAS, the wild-type sequence of PXYL2 in the plasmid YEp-KR was replaced with the corresponding sequence of pUC18-PXYL2 (CAS) to form plasmid YEp-KCAS. All other plasmids were constructed in a similar manner (Supplementary Table 2).

### Synthetic Sugar Medium

Yeast cells cultured in YPD with shaking at 200 rpm for 16 h were collected by centrifugation, washed, and used to inoculate a 500-mL flak containing fresh YPDX medium (200 mL, 10 g/L yeast extract, 20 g/L peptone, 50 g/L or 80 g/L or 100 g/L glucose and 50 g/L xylose) to an initial OD_600_ of 1 (Nambu-Nishida et al., 2018). The fermentation was performed at 30°C and 150 rpm shaking for 72 h under anaerobic conditions.

### Hydrolysates With Added Urea

The seed cultures were obtained as described above, washed with saline and re-suspended in the corn stover hydrolysate containing 1 g/L urea before inoculation (provided by the company), then fermented it at 30°C in a 500-mL flask (Mataffo et al., 2020).

### Pretreatment of Corn Flour (CF)

Using aseptic CF as substrate (30%, calculated by solid mass), sterile filtered α-amylase (0.064% of corn concentration) was added to liquefy at 85°C for 4 h (pH 5.7). Then, the amyloglucosidase (0.1% of CF concentration) used for saccharification and Novisin cellulase (0.6% of CF concentration) were added for the pre-degradation within 0.5 h at 50°C (pH 4.8), which was transferred to a flask used for yeast fermentation, seeded to an OD_600_ of 1.0 and grown at 30°C and 150 rpm shaking for 72 h. The initial pH was set to 4.6.

### Treatment of Corn Distiller’s Grains

The distiller’s grains stored at 4°C for 24 h were fermented for 96 h and treated with dilute acid (95°C, 1% dilute sulfuric acid, 90 min). The material was weighed and made up with sterile water to the previous quantity. After pre-treatment, rotary vacuum steam treatment (−0.09 MPa, 85°C, 30 min) was used to remove the remaining ethanol from the distiller’s grains. After steaming, the dry matter concentration was kept constant by adding water (Biener et al., 2012). The pH of the system was adjusted to 4.8–5.0. Afterward, the distiller’s grains were hydrolyzed with 0.6% cellulase and 1.5% xylanase at 50°C and 250 rpm shaking for 24 h (Hu et al., 2015; Xiao et al., 2019). Yeast cells were then used for fermentation at pH 4.6, 30°C and 150 rpm shaking (initial OD_600_ of 1).

### Preparation of Corncob Hydrolysates

A mixture of 0.5% (w/w) H_2_SO_4_ and 1.5% (w/w) H_3_PO_4_ was added to the crushed corncob set aside after drying at 105°C for 4 h at a solid-liquid ratio of 1:3, followed by treatment at 128°C for 1 h. Subsequently, the solid-liquid ratio and pH value were adjusted to 1:8 and 5, respectively, followed by adding 2 g/L peptone and 5 g/L yeast extract, and sterilization at 115 °C for 15 min. Seed cultures obtained by activation, amplification and culture (30°C, 150 rpm) were added to the resulting non-detoxicated hydrolysate (initial OD_620_ of 2). Then, the mixtures were subjected to two-stage simultaneous saccharification co-fermentation (SSCF) at 40°C and 200 rpm shaking for 60 h in 500-mL flask, in which cellulose (20 FPU/g) was added after first stage fermentation and fermented for 36 h sequentially (Du et al., 2020).

### Dilute Acid (DA) and Dilute Alkali (AL) Treatment of Corn Stover (CS)

The dilute acid (DA) pretreatment of corn stover was carried out in a 2-L high-pressure reactor with 10% (w/w) biomass and 1% (w/w) H_2_SO_4_ at 160°C for 10 min, and the CS slurry was neutralized with NaOH to pH 7.0 (Avci et al., 2013). The neutralized biomass was washed until the filtrate was clear, and dried at 60 °C until the moisture of CS after treatment was 10–20%. The dilute alkali (AL) pretreatment was performed in a 1-L flask containing 2% (w/w) sodium hydroxide, and 10% (w/w) biomass at 121°C for 20 min. After pretreatment, the pH of the CS slurry was adjusted to 7.0 with HCl, and the neutralized biomass was washed until the filtrate was clear, and dried at 60°C until the moisture of CS after treatment was 10–20%. All the pretreated CS was stored at 4°C until further use.

The enzymatic hydrolysis of pretreated biomass at different substrate concentrations (20 and 30%) was carried out at pH 4.8, 50°C, and 250 rpm shaking for 72 h. The total enzyme dosage was 40 mg protein/g polysaccharides, and the cellulase to xylanase ratio was 7:3. When the substrate concentration was 20 and 30%, the biomass and enzyme was fed at a ratio of 1:1 and 2:1, respectively. Sugar concentrations of the hydrolysates are shown in Supplementary Figure 2.

### Quantitative Measurements During Fermentation

Microscopy was utilized to examine whether bacteria contaminated the yeast cultures and observe the germination or mortality rate of S. cerevisiae after staining it with methylene blue. During the experiment, cells from both the logarithmic phase and the initial cell volume needed to be determined by spectrophotometry at 600 nm or 620 nm (OD_600_ or OD_620_). One mL fermentation samples were taken at indicated time under sterile conditions and each component was measured by high performance liquid chromatography (HPLC), as described previously (Cao et al., 2014).

## Results and Discussion

### Evaluation of Haploid and Diploid Strains Harboring *XR* and/or *XDH* Mutations

**FIGURE 1:**
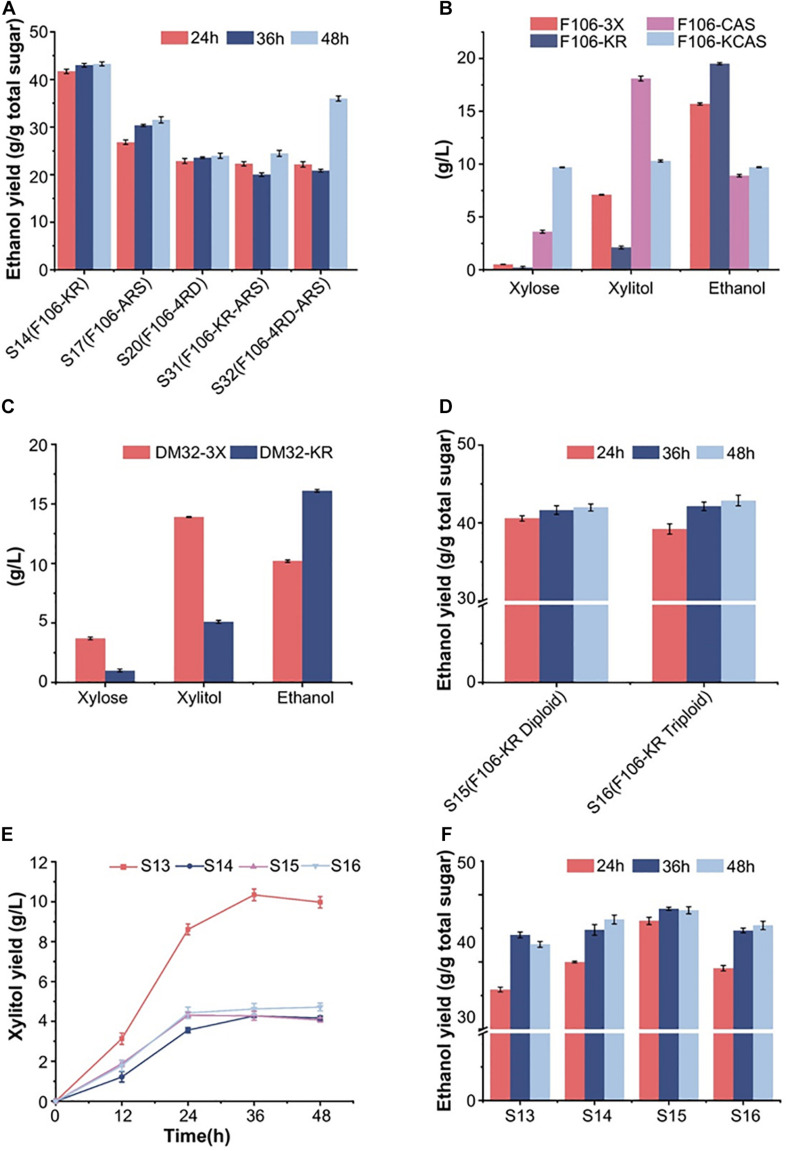
Anaerobic fermentation results of engineered yeast strains in synthetic media (glucose and/or xylose). **(A)** Ethanol production of 5 haploid strains during 48 h in mixed sugar medium (50 g/L glucose and 50 g/L xylose). **(B)** The xylose, xylitol and ethanol production of the haploid F106 carrying different plasmids (3X, KR, CAS, KCAS) after fermentation in pure xylose medium for the indicated time. **(C)** The fermentation results of diploid strains DM-3X and DM-KR for three days in a synthetic xylose medium. **(D)** Ethanol yield of S15 (F106-KR diploid) and S16 (F106-KR triploid) in a mixed sugar medium. Xylitol **(E)** and ethanol **(F)** yield of indicated strains after fermenting for 48 h in simulated corn stover hydrolysate (80 g/L glucose, 40 g/L xylose with 3 g/L acetate). The experimental results were averaged over three parallel experiments with standard deviations.

The previously reported *S. cerevisiae* haploid strain F106-KR with the XR-K270R mutation produced 77.6 g/L ethanol from 220 g/L xylose and had an ethanol yield of 0.42 g/g total sugar in mixed sugar fermentation, which makes it a highly promising industrial strain (Xiong et al., 2013). It was speculated that a redox imbalance caused by different cofactor preferences of XR and XDH leads to increased xylitol accumulation and reduced ethanol formation during fermentation, which could be alleviated by modifying the XR or XDH enzymes via protein engineering (Jeppsson et al., 2006; Watanabe et al., 2007b; Petschacher and Nidetzky, 2008; Matsushika et al., 2009). Indeed, a mutant form of ARSdR XDH with multiple mutations (D207A/I208R/F209S/N211R, or ARS), which resulted in a complete reversal of coenzyme specificity toward NADP+, was reported to increase ethanol production with decreased xylitol content (Watanabe et al., 2005, 2007b). Other *S. cerevisiae* strains harboring the NADH-preferring XR mutations, like *K274R* and *K274R/N276D* (named 4RD) from *Candida tenuis*, also showed increased ethanol yields and decreased xylitol accumulation (Petschacher et al., 2005; Petschacher and Nidetzky, 2008). Hence, we constructed five engineered strains based on strain F106, which were named S14 (F106-KR, control strain), S17 (F106-ARS), S20 (F106-4RD), S31 (F106-KR-ARS) and S32 (F106-4RD-ARS). After 48 h of fermentation, the ethanol yield of S14 (0.42 g/g) was the highest among the five mutant strains in the mixed sugar medium (50 g/L glucose and 50 g/L xylose) (Figure 1A), indicating that the XR-K270R substitution effectively addressed the redox imbalance to promote ethanol production. In addition, strain S32 with combined ARS and K274R/N276D mutations showed a better ethanol yield over time than other three strains S17, S20 and S31 (Figure 1A). The results showed that the ethanol yield of was different. We speculated that strains with combination of different *XR*- and *XDH*-related gene mutations could alter the redox imbalance and lead to differences in the ethanol production.

We obtained five XR and XDH mutant strains in previous genetic engineering research, including the efficient strain XR-K270R and the ineffective strain XDH-ARS. Here, we included the XDH-related CAS mutant strain in an attempt to minimize the xylitol production. Firstly, we selected F106 strains carrying plasmids YEp-3X (F106-3X), YEp-KR (F106-KR, S14), YEp-CAS (F106-CAS) and YEp-KCAS (F106-KCAS) to assess the effects of these mutations on xylose metabolic efficiency. In a xylose medium, the ethanol yield of S14 was 46% higher, and its xylitol production was 53% lower than those of F106-3X (Figure 1B). While no strain was able to completely consume xylose, S14 consumed more xylose than F106-3X. These results further indicate that the K270R mutant can minimize the redox imbalance and xylitol accumulation, thereby promoting ethanol production. Based on the confirmed properties of the XR-K270R mutant, we integrated pUC-3XK270R into the diploid strain YC-DM for further evaluation. Compared with DM32-3X, the ethanol yield of DM32-KR was increased by 60%, while the xylitol level was decreased by 63%, indicating that the XR-K270R mutation is more effective in the diploid yeast than in its haploid parent strain (Figures 1B,C). There are two possible explanations for the increased ethanol yield of F106-KR and DM32-KR: (1) Both haploid and diploid strains carrying the XR-K270R mutation alleviated the redox imbalance and reduced the accumulation of xylitol to some extent, resulting in an increase of ethanol production; (2) Diploid strains confer higher ethanol resistance and stronger inhibitor tolerance than haploids, and further exaggerate effects of the K270R mutation, making diploid strains more robust and suitable for industrial ethanol production than haploids. Moreover, we obtained diploid S15 and triploid S16 strains from S14. And our fermentation results show that the ethanol yield of S16 was slightly higher than S15 within 48 h (Figure 1D). The physiological characteristics of these engineered strains indicate that increased chromosomal copies improve xylose fermentation efficiency.

### Fermentation Performance of Different Strains in Simulated Corn Hydrolysates

To investigate the strain tolerance to fermentation inhibitors, we fermented the control strain S13 (F106-3X) and strains S14-16 in simulated corn hydrolysates (80 g/L glucose, 40 g/L xylose and 3 g/L acetate) (Xiong et al., 2011; Xiong et al., 2013). At 48 h, the xylitol yields of S13-16 were 9.97 g/L, 4.16 g/L, 4.06 g/L and 4.72 g/L, respectively (Figure 1E); hence, polyploid strains have significantly lower xylitol production than the haploid, indicating that the XR-K270R mutation successfully restored redox balance in these strains. Furthermore, the xylitol titers were decreased by 59.2% and 52.6%, respectively, in S15 and S16. The ethanol yields of S13-16 were 0.426, 0.463, 0.477 and 0.455 g/g total sugars at 48 h. Among them, the ethanol yields of S14, S15 and S16 were significantly higher than that of S13, and showed an increasing trend among S13-15. To our delight, the ethanol yield of S15 reached 93.9% of the theoretical value within 36 h, which is higher than the haploid S14 (Figure 1F). However, S16 did not show better fermentation performance than S15 in the presence of 3 g/L acetate.

### Fermentation Performance of S15 in Corn Hydrolysates With/Without Urea, Corn Flour Hydrolysates and Synthetic Sugars

**FIGURE 2:**
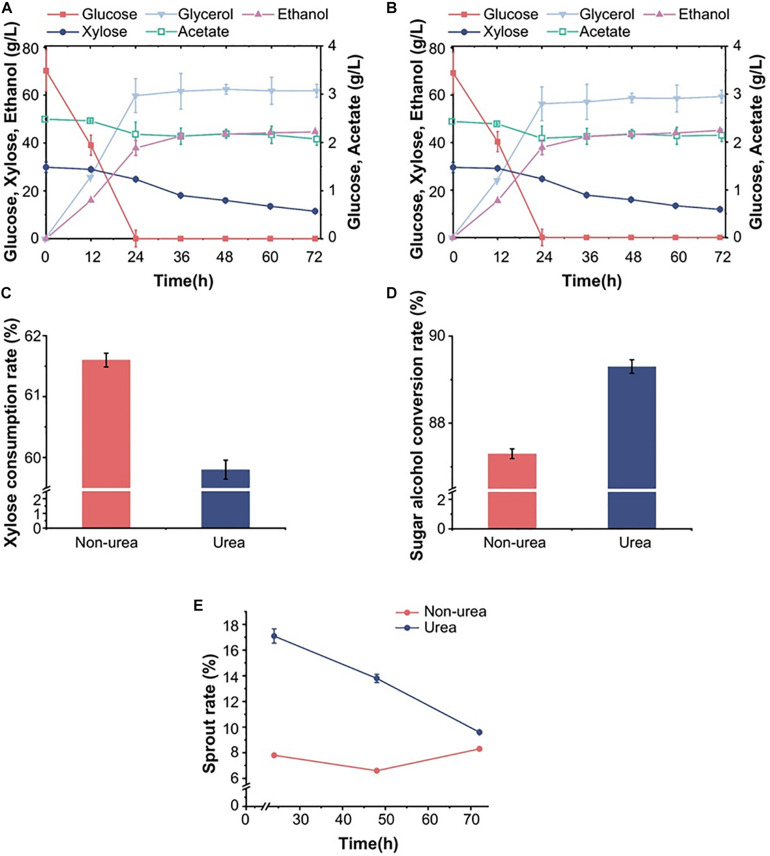
Effects of urea on the fermentation and reproduction ability of diploid strain S15 in corn stover hydrolysates. The fermentation properties of the diploid strain S15 in the hydrolysate **(A)** with and **(B)** without urea. Xylose consumption **(C)** and total sugar-to-alcohol conversion **(D)** of S15 in the two different hydrolysate-based media. **(E)** The germination rate of strain S15 under the influence of urea. The results were averaged over three parallel experiments with standard deviations.

Generally, yeast strains used in industrial fermentation are diploid, so we focused on the ability of S15 to ferment biomass hydrolysates. Similar to the fermentation performance of S14-16 in pure mixed sugars and simulated corn stover hydrolysate, S15 had better fermentation ability than S14. In view of the widespread use of diploid strains and corn stover as the raw material in the ethanol industry, we analyzed the fermentation ability of the best performing diploid S15 for 72 h using actual stover hydrolysates, and investigated the effect of urea on its fermentation efficiency. In corn stover hydrolysates with or without urea, S15 had an overall sugar-to-alcohol conversion rate of 89.3 and 87.3%, respectively (Figures 2A–D), suggesting that S15 achieved a higher ethanol yield in the presence of moderate urea, which was higher than that of the reported SFA1^OE^ strain (Zhu et al., 2020). Moreover, the cell number, the germination rate and the mortality of S15 were quantified by microscopy to assess the effects of urea. The germination rate of S15 with urea showed twofold increase over the culture without urea within 24 h, and it also exhibited an increased germination rate and reduced mortality in the presence of urea from 48 to 72 h (Figure 2E). These results indicate that urea effectively helps ethanol production by promoting yeast cell germination.

**FIGURE 3:**
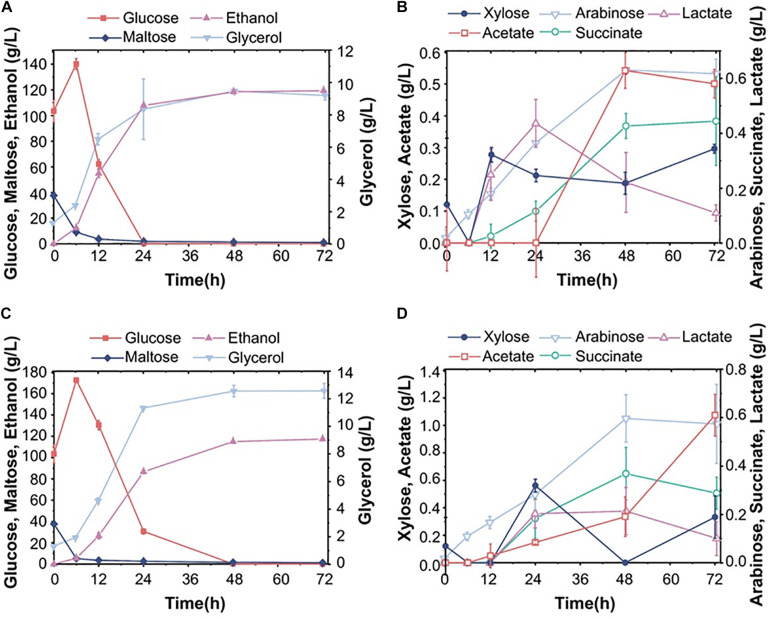
Fermentation performance of the diploid strain S15 using corn flour as raw material. **(A,B)** The fermentation performance of the industrial Angel yeast strain. **(C,D)** Fermentation of the diploid strain S15. The results were averaged over three parallel experiments with standard deviations.

**FIGURE 4:**
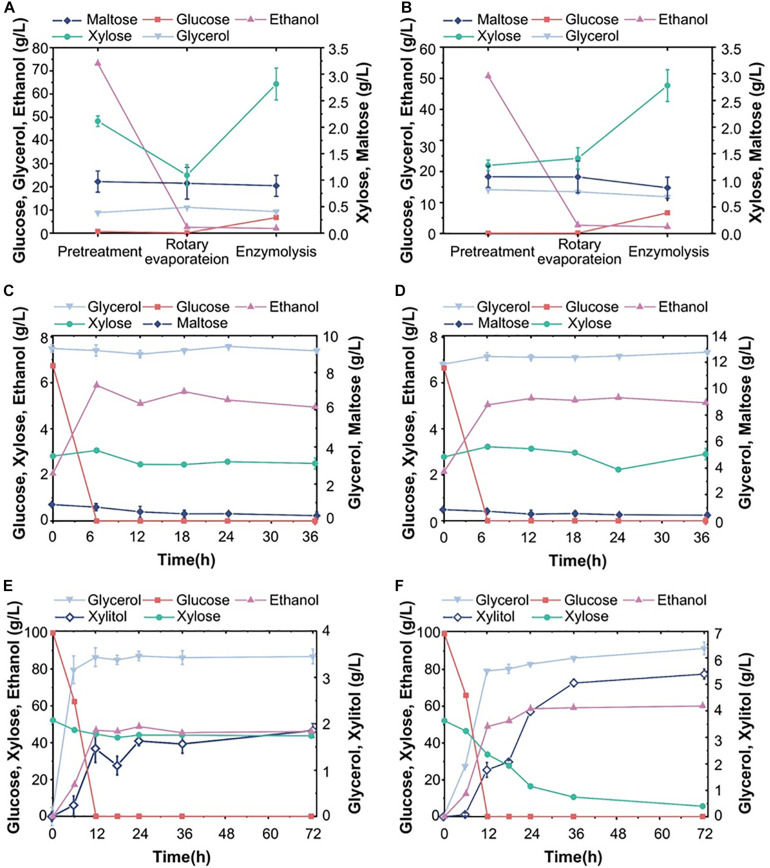
Composition of corn distiller’s grains and pure sugar medium. **(A,B)** The change in the content of each component in the fermentation system after pretreatment, rotary vacuum steam treatment and enzymatic hydrolysis, followed by fermentation with Angel yeast **(A)** and S15 **(B)**. **(C)** and **(D)** show the fermentation properties of Angel yeast and S15, respectively. **(E)** and **(F)** show the fermentation performance of Angel yeast and S15, respectively, in a synthetic medium containing 100 g/L glucose and 50 g/L xylose. The experimental results were averaged over three parallel experiments with standard deviations.

In order to evaluate the commercial value of S15, we also fermented it in parallel with the commonly used commercial strain Angel in 30% corn flour hydrolysate (mainly glucose and maltose, mass fraction). As can be seen in Figures 3A,C, the maltose concentration decreased evidently within 12 h, while the glucose content increased briefly, indicating that maltose was preferentially utilized over glucose. The Angel yeast and S15 had ethanol yields of 119.4 and 117.5 g/L, respectively. S15 also accumulated more glycerol and acetate than the control strain within 72 h (Figures 3B,D). After a comprehensive analysis, it was found that the fermentation performance of S15 was slightly lower than that of the industrial strain on corn flour hydrolysates. To overcome this, we improved the utilization rate of corn flour by treating the fermentation broth using different methods (Supplementary Figure 1). After pretreatment, rotary evaporation and enzymolysis, the ethanol level decreased dramatically and xylose increased in corn distiller’s grains (Figures 4A,B). Then, Angel yeast and S15 were grown in the processed corn distiller’s grains with less carbon sources compared to corn flour, and the two strains had similar low ethanol production (Figures 4C,D). Therefore, we speculated that a large number of by-products were generated after a series of treatments, including acid treatment, which inhibited the fermentation and yeast cell viability. In general, the utilization rate of corn flour and the conversion rate of sugar-to-ethanol were obviously improved through secondary fermentation, which achieved the goal of maximum ethanol production by two successive fermentation stages.

Subsequently, S15 and Angel yeast were fermented in a mixed sugar medium (100 g/L glucose and 50 g/L xylose) for comparative analysis. The results showed that both strains could consume all glucose within 12 h. After 72 h, S15 showed a higher xylose consumption of 46.5 g/L and produced 60.1 g/L ethanol, along with higher concentrations of xylitol and glycerol than that of Angel yeast (Figures 4E,F). These results demonstrated that S15 had a better ability to metabolize xylose and produce ethanol than Angel yeast, but the difference was not obvious in the hydrolysates.

### Analysis of Tolerance and Utilization of Corncob by Strains S15 and S16

**FIGURE 5:**
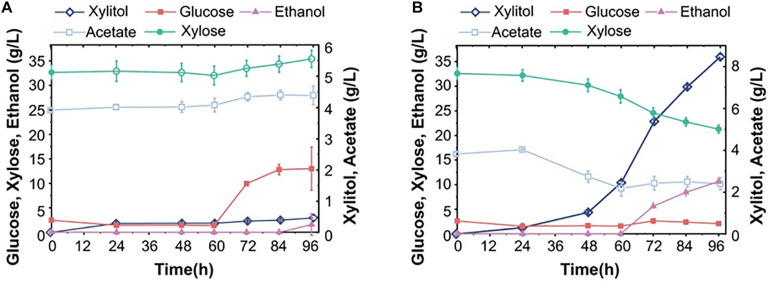
Fermentation performance of diploid and triploid strains in corncob hydrolysate-based medium. The ethanol yields of S15 **(A)** and S16 **(B)** from corncob hydrolysate were compared. The results were averaged over three parallel experiments with standard deviations.

After showing that diploid S15 had similar fermentation characteristics with the adapted industrial strains, we further explored whether the triploid S16 could produce more ethanol from corncob hydrolysates. We tested S15 and S16 in the non-detoxified corncob fermentation broth using an improved two-stage fermentation strategy at 40°C. S15 and S16 left 13.0 g/L and 2.1 g/L residual glucose while consuming 2.8 g/L and 11.3 g/L xylose within 96 h of SSCF, respectively (Figures 5A,B), showing that S16 converted monosaccharides into ethanol more efficiently than S15. It follows from above that S16 grew better than S15 at high temperature, indicating that S16 has increased temperature tolerance. Moreover, S16 consumed about 1.4 g/L acetate, while S15 produced 0.47 g/L acetate within 96 h (Figures 5A,B). Our results confirmed that S16 could produce more ethanol than S15 largely due to its lower acetate production under the same conditions. As a result, the ethanol yield of S16 was about 10.6 g/L, which was 7 times higher than that of S15.

Taken together, these results show that S16 is superior to S15, an observation consistent with the gradual increase in the performance of S14, S15 and S16 in the pure sugar fermentation medium, which is also in agreement with a recent study (Oomuro et al., 2019). We speculate that as the chromosome copy number increases, the expression of genes involved in high-temperature tolerance, acetate degradation, xylose utilization and ethanol production are increased, resulting in the enhancement of the fermentation performance in polyploid strains.

### Utilization of Acid- or Alkali-Treated Corn Stover Hydrolysates by S15 and S16

**FIGURE 6:**
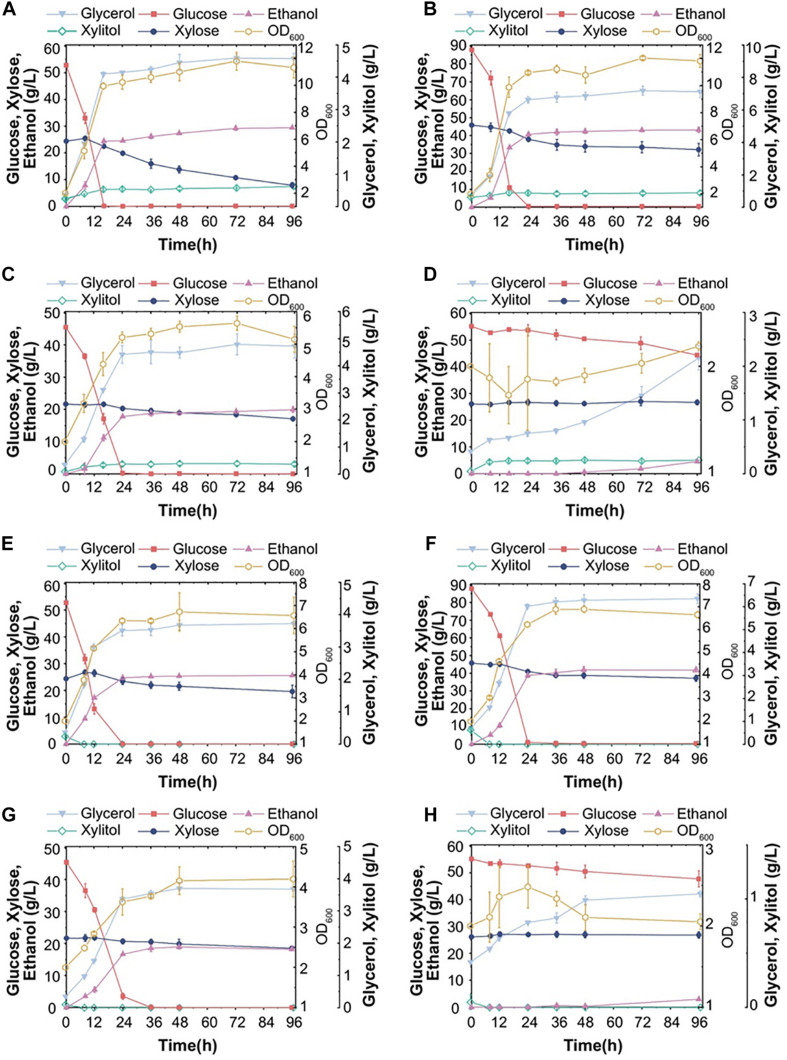
Fermentation ability of S15 and S16 with various corn stover hydrolysates. Fermentation performance of S15 in hydrolysate pretreated with dilute acid (DA) with a substrate concentration of 20% **(A)** or 30% **(B)**, and in hydrolysates pretreated with dilute alkali (AL) with a substrate concentration of 20% **(C)** or 30% **(D)**. Fermentation performance of S16 in DA-treated hydrolysates of 20% **(E)** or 30% **(F)** substrate, and in AL-treated hydrolysates of 20% **(G)** or 30% **(H)** substrate. The results were averaged over three parallel experiments with standard deviations.

Strains S15 and S16 were chosen to study whether the ethanol production in corn stover hydrolysates is positively correlated with the chromosome copy number. We used different evaluated systems with improved pretreatment methods (dilute acid or alkali treatment) and substrate concentrations (20 or 30%) to assess yeast fermentation performance. Figures 6A,B shows that S15 consumed all glucose from DA-treated hydrolysate, at both substrate concentrations of 20% and 30%; however, the corresponding xylose utilization ratios were only 67.7% and 29.9%, while the ethanol yields were 29.4 g/L and 43.2 g/L, respectively. Furthermore, the xylitol production in the hydrolysate with 30% substrate was lower than that with 20% substrate (Figures 6A,B). Hence, S15 converted more carbon source into ethanol in corn stover hydrolysates with higher substrate concentration in our experimental setting. In contrast, in the corn stover hydrolysate treated with AL, S15 consumed all glucose at a substrate concentration of 20%, compared to only 19.5% at a substrate concentration of 30% (Figures 6C,D). Moreover, the yeast cell number was several times higher in AL-treated hydrolysate with 30% substrate than that with 20% substrate, although only small amount of xylose was consumed by S15 in both AL-treated systems (Figures 6C,D). Overall, the ethanol yields from the AL-treated hydrolysates were remarkably lower than from DA-treated hydrolysates, suggesting that corn stover treated with AL contains more inhibitors than that with DA, thus limiting the fermentation capacity of S15.

Under the same experimental conditions, S16 consumed almost all glucose from DA-treated corn stover hydrolysates at both substrate concentrations, but the xylose utilization remained poor and xylitol was almost undetectable (Figures 6E,F). The ethanol production in the hydrolysate with 30% substrate was 41.81 g/L, which was higher than in 20% substrate (Figures 6E,F). The fermentation ability of S16 in AL-treated hydrolysates was lower than DA-treated hydrolysates and comparable to that of S15 (Figures 6G,H). In the AL-treated hydrolysate with 30% substrate, S16 did not consume xylose, and the number of cells remained constant (Figures 6G,H).

In summary, the maximum ethanol productions in four hydrolysates (DA 20%, DA 30%, AL 20% and AL 30%) are 76.2%, 83.3%, 78.1% and 16.3% for S15 (Figures 6A–D), and 87.3%, 85.0%, 73.3% and 10.6% for S16 (Figures 6E–H) of the theoretical value, respectively. Hence, the fermentation performance of S16 appears to be better than that of S15, particularly with DA-treated low substrate concentration.

## Conclusion

This study constructed the diploid and triploid strains (S15/S16) derived from S14 that harbors a better effective XR-K270R over other mutants with different XR and/or XDH mutation. Diploid and triploid strains were derived from the XR-K270R background, and fermentation results showed that the sugar-ethanol conversion of the diploid reached 93.9% of the theoretical value in simulated corn stover hydrolysates. Furthermore, the triploid displayed better fermentation characteristics than the diploid in hydrolysates of corncob and corn stover with different pretreatment methods (Supplementary Table 4). Compared with other studies, the polyploid strains constructed in this study also have their own advantages with rational mutants and disadvantages (Supplementary Table 5). Our results demonstrate that with the increase of chromosome copy number, the ethanol production was gradually improved, which offers basis for improving cellulosic ethanol production by genetic engineering of yeast.

## Data Availability Statement

The original contributions presented in the study are included in the article/Supplementary Material, further inquiries can be directed to the corresponding author/s.

## Author Contributions

LL did the investigation, formal analysis, and writing – original draft. MH, MJ, WY, ZX, and YK did the supervision. YZ, MK, SA, and ZJ wrote – original draft. WX did the supervision, writing - review and editing. LC did the conceptualization. All authors contributed to the article and approved the submitted version.

## Conflict of Interest

The authors declare that the research was conducted in the absence of any commercial or financial relationships that could be construed as a potential conflict of interest.

## Supplementary Material

Supplementary Figure 1 Flow chart of the whole acid treatment process from corn flour fermentation broth to corn distiller’s grains.

Supplementary Figure 2 The sugar concentration of corn stover hydrolysates with the treatment of dilute acid (DA) and dilute alkali (AL) at different substrate. concentration (20% and 30%).

Supplementary Table 1 Strains used in this study.

Supplementary Table 2 Plasmids used in this study.

Supplementary Table 3 Primers used in this study.

Supplementary Table 4 Fermentation ability of S15 and S16.

Supplementary Table 5 Advantages and disadvantages compared with other related articles.
