# Supplementary material for: Engineered Polyploid Yeast Strains Enable Efficient Xylose Utilization and Ethanol Production in Corn Hydrolysates
Source: Front Bioeng Biotechnol. 2021 Mar 5;9:655272. doi: 10.3389/fbioe.2021.655272 (PMC7973232; doi:10.3389/fbioe.2021.655272)
Supplement: Supplementary Figure 1 — Flow chart of the whole acid treatment process from corn flour fermentation broth to corn distiller’s grains. [file Data_Sheet_1.docx]

Supplementary Materials

Brief description:

Journal: *Frontiers in Bioengineering and Biotechnology*

Title: " **Engineered polyploid yeast strains enable efficient xylose utilization and ethanol production in corn hydrolysates** "

**Contents**

Supplementary Figures 1 - 2

Supplementary Tables 1 - 5

Supplementary References

**
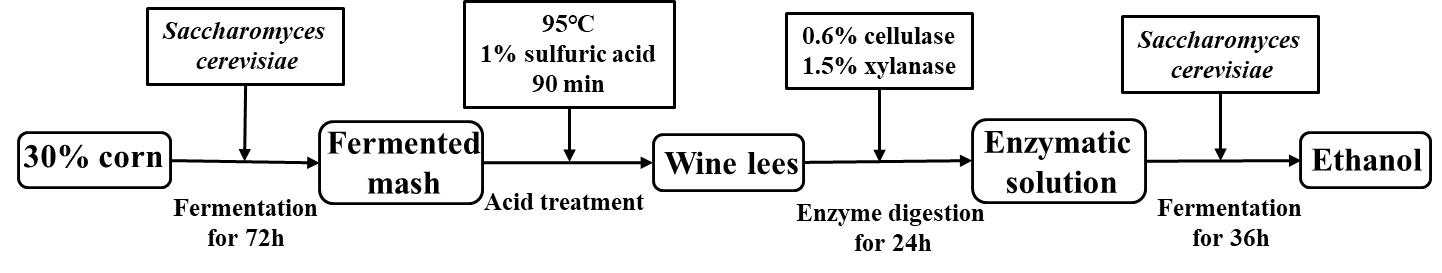
**

**Figure S1.** Flow chart of the acid treatment process from corn flour fermentation broth to corn distiller's grains.





**Figure S2.** The sugar contents in corn stover hydrolysates with treatments of dilute acid (DA) or dilute alkali (AL) at different substrate concentrations.

**Table S1**

Strains used in this study

| **Serial No.** | **Strains** | **Relevant genotypes** | **Source** |
| --- | --- | --- | --- |
| S4 | H106 | *MAT****a*** *ura3* *fps1**∆* *gpd2∆ P_PGK1_-GLN1-T_GLN1_* | (Xiong et al., 2011) |
| S12 | F106 | *MAT****a*** *ura3* *fps1∆ gpd2∆P_PGK1_*-*GLN1*-*T_GLN1_*  *P_PDC1_*-*TKL1*-*T_TKL1_*/*P_PGK1_*-*TAL1*-*T_TAL1_*/ *P_TPI1_*-*RKI1*-*T_RKI1_*/*P_ADH1_*-*RPE1*-*T_RPE1_* | (Xiong et al., 2011) |
| S13 | F106-3X | *MAT****a*** *ura3 fps1∆ gpd2∆P_PGK1_*-*GLN1*-*T_GLN1_* /  *P_PDC1_*-*TKL1*-*T_TKL1_*/*P_PGK1_*-*TAL1*-*T_TAL1_*/ *P_TPI1_*-*RKI1*-*T_RKI1_*/*P_ADH1_*-*RPE1*-*T_RPE1_*/  *P_ADH1_*-*XYL1*-*T_ADH1_*/*P_PGK1_*-*XYL2*-*T_PGK1_*/*P_PGK1_*-*XKS1*-*T_PGK1_* | (Xiong et al., 2011; Xiong et al., 2013) |
| S14 | F106-KR | *MAT****a*** ura3 *fps1∆* *gpd2∆P _PGK1_*-*GLN1*-*T_GLN1_*  *P_PDC1_*-*TKL1*-*T_TKL1_*/*P_PGK1_*-*TAL1*-*T_TAL1_*/ *P_TPI1_*-*RKI1*-*T_RKI1_*/*P_ADH1_*-*RPE1*-*T_RPE1_*/  *P**_ADH1_*-*XYL1*(K270R)-*T_ADH1_*/*P_PGK1_*-*XYL2*-*T_PGK1_*/*P_PGK1_*-*XKS1*-*T_PGK1_* | (Xiong et al., 2011) |
| S15 | F106-KR (Diploid) | *MAT****a****/α ura3* *fps1∆* *gpd2∆P_PGK1_*-*GLN1*-*T_GLN1_*  *P_PDC1_*-*TKL1*-*T_TKL1_*/*P_PGK1_*-*TAL1*-*T_TAL1_*/*P_TPI1_*-*RKI1*-*T_RKI1_*/*P_ADH1_*-*RPE1*-*T_RPE1_*/  *P_ADH1_*-*XYL1*(K270R)-*T_ADH1_*/*P_PGK1_*-*XYL2*-*T_PGK1_*/*P_PGK1_*-*XKS1*-*T_PGK1_* | This study |
| S16 | F106-KR (Triploid) | *MAT****a****/****a****/α ura3* *fps1∆ gpd2∆ P_PGK1_*-*GLN1*-*T_GLN1_*/ *P_PDC1_*-*TKL1*-*T_TKL1_*/*P_PGK1_*-*TAL1*-*T_TAL1_*/*P_TPI1_*-*RKI1*-*T_RKI1_*/*P_ADH1_*-*RPE1*-*T_RPE1_*/  *P_ADH1_*-*XYL1*(K270R)-*T_ADH1_*/*P_PGK1_*-*XYL2*-*T_PGK1_*/*P_PGK1_*-*XKS1*-*T_PGK1_* | This study |
| S17 | F106-ARS | *MAT****a*** *P_ADH1_*-*XYL1*-*T_ADH1_*/*P_PGK1_*-*XYL2*(D207A/I208R/F209S)-*T_PGK1_*/*P_PGK1_*-*XKS1*-*T_PGK1_* | This study |
| S20 | F106-4RD | *MAT****a*** *P_ADH1_*-*XYL1*(K274R/N276D)-*T_ADH1_*/*P_PGK1_*-*XYL2*-*T_PGK1_*/*P_PGK1_*-*XKS1*-*T_PGK1_* | This study |
| S31 | F106-KR-ARS | *MAT****a*** *P_ADH1_*-*XYL1*(K270R)-*T_ADH1_*/*P_PGK1_*-*XYL2*(D207A/I208R/F209S)-*T_PGK1_*/  *P_PGK1_* -*XKS1*-*T_PGK1_* | This study |
| S32 | F106-4RD-ARS | *MAT****a*** *P_ADH1_*-*XYL1*(K274R/N276D)-*T_ADH1_*/*P_PGK1_*-*XYL2*(D207A/I208R/F209S)-*T_PGK1_*/ *P_PGK1_*-*XKS1*-*T_PGK1_* | This study |
| S36 | YC-DM | *MAT****a****/**α* | Angel Yeast, China |
|  | F106-CAS | *MAT****a*** *ura3* *fps1∆* *gpd2∆ P_PGK_*_1_-*GLN1*-*T_GLN1_*  *P_PDC1_*-*TKL1*-*T_TKL1_*/*P_PGK1_*-*TAL1*-*T_TAL1_*/*P_TPI1_*-*RKI1*-*T_RKI1_*/*P_ADH1_*-*RPE1*-*T_RPE1_*/*P_ADH1_*-*XYL1*-*T_ADH1_*/ *P_PGK1_*-*XYL2*(C4dARS)-*T_PGK1_* /*P_PGK1_*-*XKS1*-*T_PGK1_* | This study |
|  | F106-KCAS | *MAT****a*** *ura3 fps1**∆* *gpd2∆ P_PGK1_*-*GLN1*-*T_GLN1_*  *P_PDC1_*-*TKL1*-*T_TKL1_*/*P_PGK1_*-*TAL1*-*T_TAL1_*/*P_TPI1_*-*RKI1*-*T_RKI1_*/*P_ADH1_*-*RPE1*-*T_RPE1_*/  *P_ADH1_*-*XYL1*(K270R)-*T_ADH1_*/*P_PGK1_*-*XYL2*(C4ARS)-*T_PGK1_*/*P_PGK1_*-*XKS1*-*T_PGK1_* | This study |
|  | DM-3X | *MAT****a****/αP_ADH1_* -*XYL1*-*T_ADH1_*/*P_PGK1_*-*XYL2*-*T_PGK1_*/*P_PGK1_*-*XKS1-T_PGK1_* | This study |
|  | DM-KR | *MAT****a****/αP_ADH1_*-*XYL1*(K270R)-*T_ADH1_*/*P_PGK1_*-*XYL2*-*T_PGK1_*/*P_PGK1_*-*XKS1*-*T_PGK1_* | This study |
|  | DM21 | *MAT****a****/α* *fps1*::REP *gpd2*::REP *P_GLN1_*-REP | This study |
|  | DM32 | *MAT****a****/α* *fps1*::REP *gpd2*::REP *P_GLN1_*-REP | This study |
|  | DM32-KR | *MAT****a****/α* *fps1*::REP *gpd2*::REP *P _GLN1_*-REP  *P_ADH1_*-*XYL1*(K270R)-*T_ADH1_*/ *P_PGK1_*-*XYL2*-*T_PGK1_*/*P_PGK1_*-*XKS1*-*T_PGK1_* | This study |

**Table S2**

Plasmids used in this study

| **Plasmids** | **Marker and description** | **Reference** |
| --- | --- | --- |
| pUC18 | AMP^R^ | (Kong et al., 2007) |
| pUC-3X | AMP^R^, *P_ADH1_*-*XYL1*-*T_ADH1_*/*P_PGK1_*-*XYL2*-*T_PGK1_*/*P_PGK1_*-*XKS1*-*T_PGK1_* | (Xiong et al., 2011) |
| pUC-3XK270R | AMP^R^, *P_ADH1_*-*XYL1*(K270R)-*T_ADH1_*/*P_PGK1_*-*XYL2*-*T_PGK1_*/*P_PGK1_*-*XKS1*-*T_PGK1_* | (Xiong et al., 2011) |
| YEp-KR | *URA3*, *P_ADH1_*-*XYL1*(K270R)-*T_ADH1_*/*P_PGK1_*-*XYL2*-*T_PGK1_*/*P_PGK1_*-*XKS1*-*T_PGK1_* | This study |
| YEp-CAS | *URA3*, *P_ADH1_*-*XYL1*-*T_ADH1_*/*P_PGK1_*-*XYL2*(C4dARS)-*T_PGK1_*/*P_PGK1_*-*XKS1*-*T_PGK1_* | This study |
| YEp-KCAS | *URA3*, *P_ADH1_*-*XYL1*(K270R)-*T_ADH1_*/*P_PGK1_*-*XYL2*(C4ARS)-*T_PGK1_*/  *P_PGK1_*-*XKS1*-*T_PGK1_* | This study |

**Table S3**

Primers used in this study

| **Primer name** | **Sequence (5’-3’)** |
| --- | --- |
| XYL2-U | GGGCCCGTCGACATGACTGCTAACCCTTCCTTGG |
| XYL2-D | GGGCCCTCTAGATTACTCAGGGCCGTCAATGA |
| XYL2KPN-U | CTAACTCCAAGGAAGGCGAA |
| XYL2KPN-D | GGGCCCGAATTCTCTAGAGGTACCTGGTGGGTTTGGTT |
| XYL2ARS-U | CTAAGGGTGTCATCGTCGTTGCTAGATCCGACAGAAAGT TGAAGATGGCCAAGGA |
| XYL2ARS-D | TCCTTGGCCATCTTCAACTTTCTGTCGGATCTAGCAACG ACGATGACACCCTTAG |

**Table S4** Fermentation ability of S15 and S16

| **Medium or hydrolysate/ Ethanol yield (Percentage of sugar-alcohol conversion to theoretical values)/ Strains** | | | | **S15** | **S16** |
| --- | --- | --- | --- | --- | --- |
| Mixed sugar medium | | | | 42.0±0.28 g/g | 42.9±0.15 g/g |
| Simulated corn stover hydrolysate | | | | 47.7±1.32 g/g | 45.5±0.98 g/g |
| Corncob hydrolysate-based medium | | | 1.6±0.03 g/L | 10.6±0.10 g/L |  |
| Corn stover hydrolysates | Pretreated with dilute acid (DA) | 20% | 29.4±2.42 g/L (76.2%) | 25.6±0.68 g/L (87.7%) |  |
|  |  | 30% | 43.2±1.34 g/L (83.3%) | 41.8±1.12 g/L  (85%) |  |
|  | Pretreated with dilute alkali (AL) | 20% | 19.9±0.74 g/L (78.1%) | 14.6±0.41 g/L (73.3%) |  |
|  |  | 30% | 4.6±0.04 g/L (16.3%) | 3.0±0.02 g/L (10.6%) |  |

**Table S5** Advantages and disadvantages compared with other releted articles

| The related references | Advantage | Disadvantage |
| --- | --- | --- |
| a. Influence of genetic background of engineered xylose‑fermenting  industrial Saccharomyces cerevisiae strains for ethanol production  from lignocellulosic hydrolysates.(Lopes et al., 2017) | The haploid and diploid strains were not only studied, but also triploid strains were studied. | The effect of specific inhibitors on the fermentation ability of strains was not clear. |
| b. Ploidy influences the functional attributes of de novo lager  yeast hybrids.(Krogerus et al., 2016) | Numerous mutant strains on XR and/or XDH were constructed from redox equilibrium. | Similar means |
| c. Metabolic engineering of a haploid strain derived from a triploid industrial  yeast for producing cellulosic ethanol.(Kim et al., 2017) | From mutant to polyploid, the content of this study is more plentiful, the strain can withstand 40 ℃ of high temperature. | The yield of cellulose ethanol was the highest during fermentation. |

**References:**

Kim, S.R., Skerker, J.M., Kong, II, Kim, H., Maurer, M.J., Zhang, G.C., et al. (2017). Metabolic engineering of a haploid strain derived from a triploid industrial yeast for producing cellulosic ethanol. *Metab. Eng.* 40**,** 176-185. doi: 10.1016/j.ymben.2017.02.006.

Kong, Q.X., Zhang, A.L., Cao, L.M., and Chen, X. (2007). Over-expressing *GLT1* in a *gpd2*Delta mutant of *Saccharomyces cerevisiae* to improve ethanol production. *Appl. Microbiol. Biotechnol.* 75(6)**,** 1361-1366. doi: 10.1007/s00253-007-0948-2.

Krogerus, K., Arvas, M., De Chiara, M., Magalhães, F., Mattinen, L., Oja, M., et al. (2016). Ploidy influences the functional attributes of de novo lager yeast hybrids. *Appl. Microbiol. Biotechnol.* 100(16)**,** 7203-7222. doi: 10.1007/s00253-016-7588-3.

Lopes, D.D., Rosa, C.A., Hector, R.E., Dien, B.S., Mertens, J.A., and Ayub, M.A.Z. (2017). Influence of genetic background of engineered xylose-fermenting industrial *Saccharomyces cerevisiae* strains for ethanol production from lignocellulosic hydrolysates. *J. Ind. Microbiol. Biotechnol.* 44(11)**,** 1575-1588. doi: 10.1007/s10295-017-1979-z.

Xiong, M., Chen, G., and Barford, J. (2011). Alteration of xylose reductase coenzyme preference to improve ethanol production by *Saccharomyces cerevisiae* from high xylose concentrations. *Bioresour. Technol.* 102(19)**,** 9206-9215. doi: 10.1016/j.biortech.2011.06.058.

Xiong, M., Woodruff, A., Tang, X., Tian, X., Zhang, J., and Cao, L. (2013). Comparative study on the mutated xylose reductase to increase ethanol production in xylose-utilizing *Saccharomyces cerevisiae* strains. *J. Taiwan Inst. Chem. Eng.* 44(4)**,** 605-610. doi: <https://doi.org/10.1016/j.jtice.2012.12.016>.
