# Supplementary figures and images for: Engineered Polyploid Yeast Strains Enable Efficient Xylose Utilization and Ethanol Production in Corn Hydrolysates
Source: Front Bioeng Biotechnol. 2021 Mar 5;9:655272. doi: 10.3389/fbioe.2021.655272 (PMC7973232; doi:10.3389/fbioe.2021.655272)

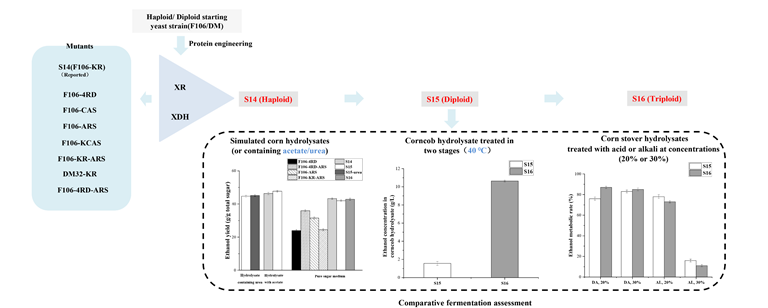

Supplement: Supplementary file 2 [file Image_1.PNG]
